# Direct Oral Anticoagulants (DOACs) are Non-Inferior to Vitamin K Antagonists for Patients Undergoing Transcatheter Aortic Valve Replacement with Indications of Anticoagulation

**DOI:** 10.31083/j.rcm2310346

**Published:** 2022-10-17

**Authors:** Jia Wang, Feng-Ying Zhang, Li Liu, Mang-Mang Pan, Chi Zhang, Jin Chen, Yuan Bian, Hou-Wen Lin, Zhi-Chun Gu

**Affiliations:** ^1^Department of Pharmacy, Ren Ji Hospital, Shanghai Jiao Tong University School of Medicine, 200127 Shanghai, China; ^2^Department of Evidence-Based Medicine and Clinical Epidemiology, West China Hospital, Sichuan University, 610041 Chengdu, Sichuan, China; ^3^Personalized Drug Therapy Key Laboratory of Sichuan Province, Department of Pharmacy, Sichuan Provincial People’s Hospital, School of Medicine, University of Electronic Science and Technology of China, 610072 Chengdu, Sichuan, China; ^4^Drug Clinical Comprehensive Evaluation Group, Shanghai Pharmaceutical Association, 200040 Shanghai, China

**Keywords:** valve replacement, anticoagulation, edoxaban, apixaban, thromboembolism, hemorrhage

## Abstract

**Background:**

The best anticoagulation choice for patients undergoing transcatheter aortic valve replacement (TAVR) with indications of oral anticoagulation (OAC) remains uncertain. We carried out a comprehensive analysis adopting updated evidence that investigated the efficacy and safety of direct oral anticoagulants (DOACs) versus vitamin K antagonists (VKAs) in this population.

**Methods:**

A systematic search has been conducted through PubMed, Embase, and Cochrane Library to collect randomized controlled trials (RCTs) and real-world studies comparing the therapy outcomes of DOACs with VKAs in patients undergoing TAVR with indications of OAC up to Dec 2021. Included studies reported all-cause mortality, bleeding, stroke, or composite endpoint. A random-effects model was used and followed a sensitivity analysis based on the heterogeneity. In addition, five scenario analyses were performed to robust our findings.

**Results:**

Our analysis included 11 articles enrolling a total of 8934 patients undergone TAVR with indications of OAC (DOACs group = 3890, VKAs group = 5044). Pooled analysis revealed no significant different risk of all-cause mortality (aHR: 0.95, 95% CI: 0.65–1.39, *$I^{2}$*: 90.6%), stroke (aHR: 0.86, 95% CI: 0.55–1.35, *$I^{2}$*: 44.3%), bleeding (aHR: 0.83, 95% CI: 0.61–1.13, *$I^{2}$*: 76.3%), and composite endpoint (aHR: 1.05, 95% CI: 0.88–1.24, *$I^{2}$*: 11.7%) in the DOACs and VKAs groups. Various forms of death, stroke and bleeding, including cardiovascular death (aHR: 0.92, 95% CI: 0.64–1.33, *$I^{2}$*: 34.1%), hemorrhagic stroke (aHR: 0.63, 95% CI: 0.23–1.75, *$I^{2}$*: 22.7%), ischemic stroke (aHR: 0.79, 95% CI: 0.56–1.15, *$I^{2}$*: 0.0%), transient ischemic attack (aHR: 0.75, 95% CI: 0.40–1.41, *$I^{2}$*: 0.0%), major or life-threatening bleeding (aHR: 0.96, 95% CI: 0.74–1.24, *$I^{2}$*: 27.9%), and minor bleeding (aHR: 0.90, 95% CI: 0.52–1.57, *$I^{2}$*: 54.3%), also showed similar rates among DOACs and VKAs groups. The results based on five scenarios confirmed the said findings.

**Conclusions:**

Compared with VKAs, the efficacy and safety of DOACs were comparable for treating TAVR patients combined with anticoagulation indications. Further large-scale RCTs investigating more detailed scenarios are still needed to confirm the optimal anticoagulation strategy.

## 1. Introduction

Transcatheter aortic valve replacement (TAVR) is an increasingly used procedure for patients with severe aortic stenosis (AS), which is considered the preferred strategy in inoperable or high-risk patients [1, 2]. The updated European guidelines recommend that single antiplatelet therapy should be treated for life after TAVR if there is no evidence of anticoagulation. However, lifelong oral anticoagulation (OAC) is recommended for TAVR patients with anticoagulation indications [3, 4]. Direct oral anticoagulants (DOACs) have proven identical efficacy and safety to vitamin K antagonists (VKAs) and provided new anticoagulant strategies for patients with atrial fibrillation (AF) [5]. Nevertheless, DOACs, or VKAs, the optimal anticoagulation strategy for TAVR patients needing OAC, remained elucidated. One meta-analysis revealed DOACs are non-inferior to VKAs in patients undergoing TAVR, which were limited by only including retrospective cohort studies and reporting unadjusted pooled odds ratios [6]. On the other hand, a recent meta-analysis showed VKAs are more protective in disabling or non-disabling stroke for post-TAVR patients requiring anticoagulation than DOACs [7]. However, the randomized controlled trial (RCT) included was not eligible for inclusion criteria due to applying antiplatelet therapy as control [8], possibly leading to untenable results. Meanwhile, two pivotal randomized controlled trials, ENVISAGE-TAVI and ATLANTIS [9, 10], concerning this issue have been completed, and an updated study is warranted. Therefore, we conducted a pooled analysis by summarizing all available evidence from RCTs and real-world studies comparing DOACs and VKAs in post-TAVI patients requiring anticoagulation therapy.

## 2. Methods

### 2.1 Data Source and Literature Screening

The present pooled analysis was conducted according to the Preferred Reporting Items for Systematic Reviews and Meta-Analyses (PRISMA) guidelines [11]. We searched for relevant publications on the PubMed, Cochrane Library, and Embase databases from inception to Dec 29, 2021. A flowchart of the search strategy is presented in **Supplementary Table 1**. Moreover, the data not reported in articles were searched through the ClinicalTrials. Reference lists of identified articles were also reviewed to find potential studies that met inclusion criteria. Two reviewers (J.W. and F.Z.) retrieved the eligible documents independently from databases and resolved the differences through consulting with a third author (Z.G.).

### 2.2 Selection Criteria and Study Outcomes

Inclusion criteria were as follows: (I) the study design was a retrospective, prospective cohort study, or RCT; (Ⅱ) studies with patients who underwent TAVR and in need of OAC; (III) enrolled participants were distributed to intervention group (using at least one of DOACs) and control group (using at least one of VKAs); (IV) the reported outcomes included either all-cause mortality, any stroke, any bleeding, or composite endpoint. Moreover, trials absenting control or interventional groups, case-control or cross-sectional studies, and studies lacking baseline data or insufficient efficacy and safety outcomes were excluded. The included observational studies have eliminated the patients with absolute contraindication on using DOACs, including mechanical valves, estimated glomerular filtration rates <30 mL/min/1.73 $m^{2}$, and moderate to severe mitral valve stenosis. Primary outcomes were all-cause mortality, bleeding, stroke, and composite endpoint. Since death, bleeding and stroke could be subdivided into more specific items, we also analyzed cardiovascular death, major or life-threatening bleeding, minor bleeding, hemorrhagic stroke, ischemic stroke, and transient ischemic attack (TIA). The criteria definition of major, life-threatening, or minor bleeding, and composite endpoint for each study are presented in **Supplementary Table 2**. Two reviewers (J.W. and L.L.) independently screened the titles and abstracts and filtered the full text of potentially relevant studies. Any disagreements between the two reviewers were resolved by a third investigator (Z.G.).

### 2.3 Data Extraction, Quality and Risk of Bias Assessments

Two investigators (J.W. and F.Z.) extracted data independently. Detailed data obtained from the retrieved studies includes the following sections: (I) study characteristics; (Ⅱ) patient demographics; (III) clinical characteristics; (IV) data of recorded outcomes. The Cochrane Risk of Bias Tool was used for quality assessment of included RCTs, containing the following domains: random sequence generation; allocation concealment; blinding of participants and personnel; blinding of outcome assessors; incomplete outcome data; selective reporting; and other forms of bias [12, 13]. The Newcastle-Ottawa scale (NOS) system was applied to evaluate each included real-world study. According to the NOS scoring criterion, 9 points were the maximum score, ≥7 points were considered high-level quality, 5–6 points were thought of moderate-level, and <5 points were low-level quality [14]. In addition, funnel plots, Begg’s test, and Egger’s test were used for the evaluation of potential publication bias [15, 16].

### 2.4 Data Analysis

Results for primary analysis were treated as dichotomous data. Adjusted hazard ratios (aHRs) and 95% confidence intervals (CIs) were calculated using random-effects models. Heterogeneity was tested adopting the *$I^{2}$* statistic (*$I^{2}$*>50% represents significance) [17]. Further scenario analyses were executed to check the robustness of our findings. Scenario 1: we examined the pooled relative risks (RRs) of outcomes using crude data considering some studies did not present adjusted or matched results; scenario 2: additional analyses were conducted by restricting the population to patients with the anticoagulant indication of AF; scenario 3: we performed the analysis by adding the ATLANTIS trial which was only reported at the American College of Cardiology (ACC) 2021 virtual meeting and detailed data was not available; scenario 4: further analyses were performed to estimate the effect after excluding articles with follow-up <1 year. scenario 5: we excluded the studies that did not adjust their raw data to minimize the bias of different criteria used to choose the anticoagulants. Meta-regression analysis was carried out to explore heterogeneity sources [18]. Sensitivity analyses were performed to evaluate the influence of each study by omitting one study at a time. All meta-analyses were conducted using STATA statistical software, Release 13 (Statacorp, College Station, TX, USA). Furthermore, *p *< 0.05 suggested statistically significant.

## 3. Results

### 3.1 Search Results and Study Evaluation

**Fig. 1.**
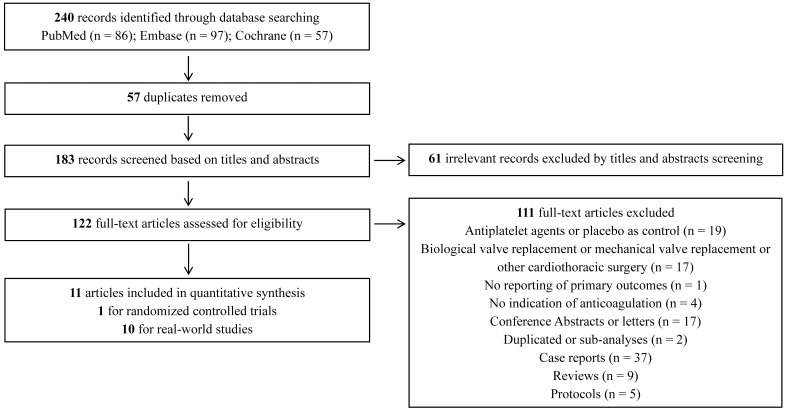
**Flow diagram for the selection of eligible studies**.

Our analysis included 1 RCT and 10 real-world studies that involved 8934 TAVR participants with anticoagulant indications, divided into the DOACs (n = 3890) or VKAs groups (n = 5044) [10]. The details of the article selection are presented in Fig. 1. The characteristics of the eligible studies are listed in Tables 1,2. For RCT [10], 713 patients received edoxaban, and 713 received VKAs. The follow-up was 2 years. Among the 10 real-world studies [19, 20, 21, 22, 23, 24, 25, 26, 27, 28], 3177 patients took DOACs, and 4331 patients took VKAs. Among them, 3 studies applied propensity-score matching (PSM) to adjust for the difference in baseline, 2 used inverse probability of treatment weighting (IPTW), 1 used Cox regression model, and the rest 4 did not adopt any adjustment methods. Publication periods were from 2018 to 2021, and the follow-up period spanned from 0.25 years to 3 years. Furthermore, 7 studies included the population with the OAC indications for AF; the other 4 studies without specified OAC indications. Patient demographics of 11 studies are outlined in** Supplementary Table 3**. The quality assessment of all studies was identified as modest to high (**Supplementary Tables 4 and 5**). We considered the quality of RCT moderate due to the open-labeled study design. For real-world studies, the NOS score of each study ≥6 points indicates modest to high quality.

**Table 1.** **Characteristics of included randomized trials**.

Study (year)	NCT number	Intervention with dosage	Patients (number)	Comparison	Patients (number)	Follow up (year)	Indication for OAC
ENVISAGE-TAVI 2021	NCT02943785	Edoxaban 60 mg once	713	Vitamin K Antagonist	713	2.0	Atrial fibrillation

OAC, oral anticoagulation; ENVISAGE-TAVI, Compare the efficacy and safety of edoxaban with vitamin K antagonists in patients with atrial fibrillation as the indication for oral anticoagulation after successful transcatheter aortic valve replacement.

**Table 2.** **Characteristics of included real-world studies**.

Study (year)	Country or region	Data source	Inclusion period	Intervention/Numbers	Comparison/Numbers	Adjusted method	Adjusted variables	Follow up (year)	Indication for OAC	Numbers of each DOAC
Didier *et al*. 2021 [19]	France	Single-payer national health data system	2010.1–2017.12	DOACs/1378	VKAs/1093	PSM	(1)	3.0	Different indications	Api/724; Riva/488; Dabi/166
Kawashima *et al*. 2020 [20]	Japan	Optimized transcatheter valvular intervention registry	2013.10–2017.5	DOACs/227	VKAs/176	IPTW	(2)	2.0	AF	NR
Mannacio *et al*. 2020 [21]	Italy	A retrospective, multicentre, cohort study	2013.7–2019.12	DOACs/340	Warfarin/692	PSM	(3)	2.7	AF	NR
Kalogeras *et al*. 2019 [22]	Athens, Tokyo, London, and Hammersmith	Athens–Tokyo–London-Aortic-Stenosis (ATLAS) registry	NR	DOACs/115	Warfarin/102	PSM	(4)	2.0	Different indications	NR
Jochheim *et al*. 2019 [23]	Europe	An investigatorinitiated multicenter observational registry study	2007.6–2017.4	DOACs/326	VKAs/636	IPTW	(5)	1.0	Different indications	Riva/175; Api/128; Dabi/23
Kosmidou *et al*. 2019 [24]	The United States and Canada	Randomized PARTNER II (Placement of Aortic Transcatheter Valve II) trial and associated registries	NR	DOACs/155	Warfarin/778	NR	NR	2.0	AF	NR
Butt *et al*. 2019 [25]	Denmark	Danish healthcare system	2012.1–2017.6	DOACs/213	VKAs/516	COX	(6)	1.0	AF	NR
Mangner *et al*. 2018 [26]	Germany	A retrospective cohort study	2011.1–2016.3	DOACs/182	VKAs/115	NR	NR	0.25	AF	Riva/111; Api/41; Dabi/29; Edo/1
Geis *et al*. 2018 [27]	Germany	A retrospective cohort study	2008.7–2017.4	DOACs/154	VKAs/172	NR	NR	0.5	Different indications	Riva/79; Api/54; Dabi/14; Edo/7
Seeger *et al*. 2017 [28]	Germany	A prospective cohort study	NR	Api/81	VKAs/50	NR	NR	1.0	AF	Api/81

DOACs, Direct Oral Anticoagulants; VKAs, Vitamin K Antagonist; COX, Cox proportional hazards models; AF, atrial fibrillation; OAC, oral anticoagulation; NR, not reported; IPTW, inverse probability of treatment weights of propensity scores; PSM, propensity score matching. (1): Adjusted variables including age, sex, body mass index, diabetes, New York Heart Association functional class III and IV, prior coronary artery bypass graft, prior percutaneous coronary intervention, prior stroke, peripheral artery disease, pacemaker, chronic renal failure, atrial fibrillation, ejection fraction, valve-in-valve procedure, aspirin, and year of inclusion in the registries; (2): Adjusted variables including body mass index, HAS-BLED score, Society of Thoracic Surgeons score, New York Heart Association functional class III or IV symptoms, history of stroke and coronary artery disease, year of TAVR procedure, access site, implanted transcatheter aortic valve type, antiplatelet regimen at discharge, and anticoagulant choice per center; (3): Adjusted variables including sex, age, body mass index, hypertension, diabetes, renal disease, liver disease, history of stroke, bleeding or myocardial infarction, previous paroxysmal or persistent AF, CHA2DS2-VASc Score, HAS-BLED Score, EURO Score II, left atrium enlargemen, poor left ventricular ejection fraction, left ventricular hypertrophy, left ventricle dilatation, native aortic valve disease, aortic bioprosthesis size, prosthesis-patients mismatch, adherence to therapy and follow-up length; (4): Adjusted variables including age, gender, smoking status, previous cardiac surgery, left ventricular ejection fraction, logistic euroscore, post procedural aortic regurgitation, type of valve and use of dual antiplatelet therapy post procedure; (5): Adjusted variables including center, year of TAVR procedure, patient age at TAVR, gender, incidence of prior transcatheter aortic valve replacement, diagnosis of chronic kidney disease, Society of Thoracic Surgeons Predicted Risk of Mortality score, left ventricular function, and prosthesis type; (6) Adjusted variables for all-cause mortality including age, sex, a history of arterial thromboembolism, ischaemic heart disease, heart failure, hypertension, peripheral arterial disease, diabetes, chronic kidney disease, liver disease, and antiplatelet therapy, adjusted variables for bleeding including the components of the modified HAS-BLED score and sex; Riva, rivaroxaban; Api, apixaban; Dabi, dabigatran; Edo, edoxaban.

### 3.2 Primary Analyses

**Fig. 2.**
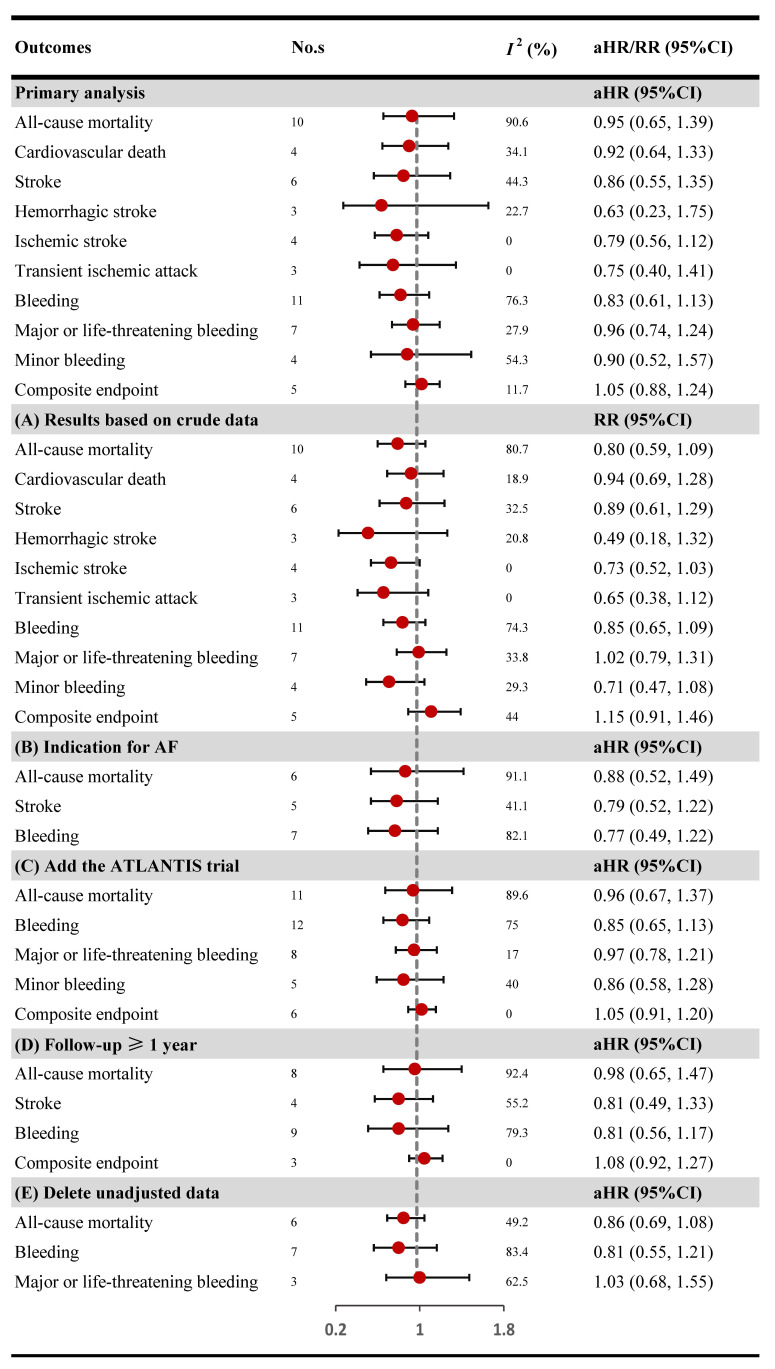
**Primary analysis and scenario analyses**. CI, confidence interval; aHR, adjusted hazard ratios; RR, relative risk. ATLANTIS: a multicenter, randomized, phase IIIb, prospective, open-label, superiority study comparing standard of care versus an apixaban-based strategy after successful TAVI. Part A: scenario analysis by calculating RR based on crude data. Part B: scenario analysis by limiting patients with anticoagulant indication as AF. Part C: scenario analysis by adding the ATLANTIS trial. Part D: scenario analysis by excluding studies with follow-up < 1 year. Part E: scenario analysis by deleting studies without adjusted data.

The pooled results indicated similar risk of all-cause mortality (aHR: 0.95, 95% CI: 0.65–1.39, *$I^{2}$*: 90.6%), stroke (aHR: 0.86, 95% CI: 0.55–1.35, *$I^{2}$*: 44.3%), bleeding (aHR: 0.83, 95% CI: 0.61–1.13, *$I^{2}$*: 76.3%) and composite endpoint (aHR: 1.05, 95% CI: 0.88–1.24, *$I^{2}$*: 11.7%) between the DOACs and VKAs groups (Fig. 2). Various forms of stroke or death which include hemorrhagic stroke, ischemic stroke, transient ischemic attack, and cardiovascular death were also showed no statistical difference among DOACs and VKAs groups (aHR: 0.63, 95% CI: 0.23–1.75, *$I^{2}$*: 22.7%; aHR: 0.79, 95% CI: 0.56–1.15, *$I^{2}$*: 0.0%; aHR: 0.75, 95% CI: 0.40–1.41, *$I^{2}$*: 0.0%; and aHR: 0.92, 95% CI: 0.64–1.33, *$I^{2}$*: 34.1%; respectively). Meanwhile, no considerable differences were also observed with regard to major or life-threatening bleeding (aHR: 0.96, 95% CI: 0.74–1.24, *$I^{2}$*: 27.9%) and minor bleeding risk (aHR: 0.90, 95% CI: 0.52–1.57, *$I^{2}$*: 54.3%) between two groups (Fig. 2).

### 3.3 Scenarios and Sensitivity Analyses

The results of scenarios analyses are presented as follows (Fig. 2). (I): the pooled RRs and associated 95% CI s of all-cause mortality (RR: 0.80, 95% CI: 0.59–1.09, *$I^{2}$*: 80.7%), stroke (RR: 0.89, 95% CI: 0.61–1.29, *$I^{2}$*: 32.5%), bleeding (RR: 0.85, 95% CI: 0.65–1.09, *$I^{2}$*: 74.3%), and composite endpoint (RR: 1.15, 95% CI: 0.91–1.46, *$I^{2}$*: 44%) based on crude data were not associated with a significantly different compared DOACs with VKAs; (II): we found the risk of all-cause mortality (aHR: 0.88, 95% CI: 0.52–1.49, *$I^{2}$*: 91.1%), stroke (aHR: 0.79, 95% CI: 0.52–1.22, *$I^{2}$*: 41.1%), and bleeding (aHR: 0.77, 95% CI: 0.49–1.22, *$I^{2}$*: 82.1%) followed restricting the population to patients in need of OAC due to AF was consistent with the main findings; (III): the pooled results of all-cause mortality (aHR: 0.96, 95% CI: 0.67–1.37, *$I^{2}$*: 89.6%), bleeding (aHR: 0.85, 95% CI: 0.65–1.13, *$I^{2}$*: 75.0%), major or life-threatening bleeding (aHR: 0.97, 95% CI: 0.78–1.21, *$I^{2}$*: 17.0%), minor bleeding (aHR: 0.86, 95% CI: 0.58–1.28, *$I^{2}$*: 40.0%), and composite endpoint (aHR: 1.05, 95% CI: 0.91–1.20, *$I^{2}$*: 0%) stayed the same by adding the up-to-date ATLANTIS trial; (IV): after excluding studies with follow-up <1 year, the results of all-cause mortality (aHR: 0.98, 95% CI: 0.65–1.47, *$I^{2}$*: 92.4%), stroke (aHR: 0.81, 95% CI: 0.49–1.33, *$I^{2}$*: 55.2%), bleeding (aHR: 0.81, 95% CI: 0.56–1.17, *$I^{2}$*: 79.3%), and composite endpoint (aHR: 1.08, 95% CI: 0.92–1.27, *$I^{2}$*: 0%) showed no statiaticl different among DOACs and VKAs. (V): the pooled results of all-cause mortality (aHR: 0.86, 95% CI: 0.69–1.08, *$I^{2}$*: 49.2%), bleeding (aHR: 0.81, 95% CI: 0.55–1.21, *$I^{2}$*: 83.4%), and major or life-threatening bleeding (aHR: 1.03, 95% CI: 0.68–1.55, *$I^{2}$*: 62.5%) were in accordance with the primary analysis after deleting unadjusted data. Sensitivity analyses revealed that no single study had s signifcant effect on the overall result (**Supplementary Table 6**).

### 3.4 Meta-Regression and Publication Bias

We next examined the potential confounding factors that may impact the all-cause mortality, stroke, bleeding, and composite endpoint outcome. Factors including mean age, percentage of females, body-mass index (BMI), hypertension, diabetes mellitus, chronic kidney disease, New York Heart Association (NYHA) class >II, history of stroke/transient ischemic attack, history of coronary artery disease, history of myocardial infarction, CHA2DS2-VASc score, and HAS-BLED score were included in the analysis. The meta-regression results displayed that the abovementioned factors did not give rise to heterogeneity (**Supplementary Table 7**). Moreover, The qualitative funnel plots indicated that included studies were absent from publication bias; Begg’s and Egger’s tests reconfirmed no significant asymmetry (*p*> 0.05) (**Supplementary Fig. 1**).

## 4. Discussion

We performed a comprehensive pooled analysis that simultaneously involves RCTs and real-world studies. The main findings can be summarized that the risk of all-cause mortality, stroke, bleeding, and composite endpoint was comparable between the DOACs and VKAs groups for post-TAVR patients requiring OAC therapy. Meanwhile, the rate of cardiovascular death, hemorrhagic stroke, ischemic stroke, transient ischemic attack, major or life-threatening, and minor bleeding was consistent between the two groups.

Compared to traditional anticoagulant VKAs, DOACs, including dabigatran, rivaroxaban, apixaban, and edoxaban, have provided an alternative therapy for venous thromboembolism and non-valvular atrial fibrillation due to their ease of use and proven efficacy and safety [29, 30, 31, 32]. Would a patient taking DOACs concurrent with valve heart disease (VHD) requires a return to VKAs is the choice we are facing? Regrettably, the results of RE-ALIGN trial were terrible, revealing excessive thromboembolism and bleeding events in patients treated with dabigatran; therefore, patients with mechanical heart valves (MHV) are contraindicated to take DOACs after the trial [33]. However, the mechanisms causing thromboembolic complications differed in MHV and bioprosthetic valves (BHV), and the latter considered less contact phase activation and device-related thrombosis [34]. A novel BHV technology, TAVR has gained popularity in patients with severe symptomatic AS [1]. The risk of thromboembolic events was increased when TAVR concomitant indications for anticoagulation. Therefore, an increasing interest in investigating the safety and efficacy of DOACs in post-TAVR patients in need of OAC therapy is arising. According to 2021 ESC/EACTS guidelines for managing valvular heart disease and 2021 ESC management of antithrombotic therapy in patients undergoing TAVR, lifelong OAC for TAVR patients who have other indications for anticoagulation were recommended intensively [3, 4]. However, robust evidence of the preferred oral anticoagulant agent for this population has not yet been established.

The initial study explored the safety and efficacy of the DOAC (apixaban) in patients with AF after TAVR was published in 2016 [28]. After that, cohort studies concerning this hot issue were conducted consecutively [26, 27, 28]. A large multicenter French TAVR registries demonstrated lower long-term mortality and major bleeding at 3 years with DOACs than VKAs at discharge [19]. An Athens–Tokyo–London-Aortic-Stenosis (ATLAS) registry showed DOACs use in patients who underwent TAVR with indication for OAC has a comparable risk of all-cause mortality and bleeding with VKAs [22]. However, all of the above studies had the drawbacks of an open-label registry treatment and controversial conclusions. Meanwhile, high-quality meta-analyses are currently lacking. The earliest meta-analysis, which combined 5 articles of 2569 patients, indicated a similar all-cause mortality, major and/or life-threatening bleeding, and stroke risk of DOACs compared with VKAs [6] (odds ratio [OR]: 1.07, 95% CI: 0.73–1.57; OR: 0.85, 95% CI: 0.64–1.12; OR: 1.52, 95% CI: 0.93–2.48, respectively). However, this study had 2 important limitations: first, only retrospective observational studies were considered, and the pooled ORs were unadjusted. Therefore, the findings should be elucidated cautiously due to the possible confounders. Second, the different indications for anticoagulation, especially AF, have not been analyzed independently. Another meta-analysis of 7 studies reported that VKAs have priority against DOACs in anti-thromboembolism (RR: 1.44, 95% CI: 1.05–1.99) but not in mortality or bleeding events [7]. Although this study was the first meta-analysis that included RCT, a severe error can not be neglected: the GALILEO trial, which compared the DOACs with antiplatelet-based therapy, did not meet the inclusion eligibility criteria [8]. Beyond that, it is noteworthy that two pivotal RCTs, ATLANTIS (NCT02664649) and ENVISAGE-TAVI (NCT02943785) [9, 10], have been reported recently. The issue could be interpreted by obtaining more valuable information on large populations. Therefore, we updated a comprehensive analysis to unite current proof from RCTs and real-world studies to judge the safety and efficacy of DOACs in the population undergoing TAVR and requiring OAC therapy. The final results revealed that using DOACs might be noninferiority to using VKAs in this setting.

We noticed *$I^{2}$* of all-cause mortality, bleeding, and stroke of primary outcomes was 90.6%, 76.3%, 44.3%, respectively, which demonstrated a substantial or mild heterogeneity across the included articles. Therefore, a meta-regression was followed to determine the heterogeneity sources, the below parameters: age, gender, BMI, hypertension, diabetes mellitus, chronic kidney disease, NYHA class, history of stroke/transient ischemic attack, history of coronary artery disease, history of myocardial infarction, CHA2DS2-VASc score, and HAS-BLED score were taken into consideration. Finally, no significant covariates were associated with the augment of heterogeneity. Consequently, the complication diseases of participants, drug adherence, and outcome measurement across studies that differ in design might account for the present heterogeneity [35].

Although we first included the high-quality data and larger population from ENVISAGE-TAVI and ATLANTIS trials, the findings of this study should be guided for clinical applications within specific situations. The ENVISAGE-TAVI trial only involved a population of older adults undergoing OAC indication for AF. The results showed edoxaban was comparable to VKAs in terms of the composite primary outcome of adverse clinical events but presented a higher risk of major bleeding than VKAs; hence, these results may not be appropriate for younger patients with low surgical risk, patients with asymptomatic aortic stenosis, patients with high bleeding risk, and those committed to other OAC indications. On the other hand, the ATLANTIS trial found no significant differences in primary, secondary, or safety endpoints among apixaban and control groups in patients with indications for anticoagulant therapy, which was a post hoc analysis results without baseline characteristics and limited population. Therefore, more extensive RCTs are needed to confirm the efficacy and safety of dabigatran and rivaroxaban in post-TAVR patients with OAC needs.

Currently, several limitations should be considered in this research. First, the outcome evaluation of individual DOACs regimens was not performed. Second, the outcome for valve thrombosis, an important concern after TAVR, was not obtained. Besides, baseline data on critical clinical parameters, such as left ventricular ejection fraction, smoking habits, alcohol consumption, and data on procedural characteristics and complications concerning the TAVR procedure were not available, which might contribute to the mild to high heterogeneity between studies. Moreover, more detailed settings, including TAVR patients with OAC indications other than AF, prior AF before TAVR, and new-onset AF after TAVR, were not performed due to a lack of adequate data. Also, we can not provide results in patients concomitant with antiplatelet regimens, whether one antiplatelet drug or dual antiplatelet therapy (DAPT), between the DOACs and VKAs due to the paucity of available data. However, we noticed exploratory results on the post hoc analysis of the ENVISAGE-TAVI trial, which showed a higher bleeding rate of edoxaban in patients combined with antiplatelet therapy than VKAs. Large RCTs are warranted to establish the clinical outcomes of DOACs when compared with VKAs in combination with antiplatelets.

## 5. Conclusions

In TAVR patients with indication of OAC, the present study indicates that DOACs are as safe and effective as VKAs in term of all-cause mortality, bleeding, stroke, and composite endpoint. However, the ideal anticoagulation scheme should be chosen through a comprehensive evaluation of the patient’s condition and the physician’s discretion. Further precise randomized controlled trials are needed to explore more scenarios, such as single DOACs regimens and combination antiplatelet therapy.

## Supplementary Material

Supplementary material associated with this article can be found, in the online version, at https://doi.org/10.31083/j.rcm2310346.
