# Supplementary material for: Direct Oral Anticoagulants (DOACs) are Non-Inferior to Vitamin K Antagonists for Patients Undergoing Transcatheter Aortic Valve Replacement with Indications of Anticoagulation
Source: Rev Cardiovasc Med. 2022 Oct 17;23(10):346. doi: 10.31083/j.rcm2310346 (PMC11267358; doi:10.31083/j.rcm2310346)
Supplement: Supplementary file 1 [file 2153-8174-23-10-346-s1.docx]

Supplementary Table 1. Search strategy.

| **Literature databases** | **Search items** | **Items found** |
| --- | --- | --- |
| PUBMED | dabigatran[Title/Abstract] OR Pradaxa[Title/Abstract] OR rivaroxaban[Title/Abstract] OR Xarelto[Title/Abstract] OR apixaban[Title/Abstract] OR Eliquis[Title/Abstract] OR edoxaban[Title/Abstract] OR Savaysa[Title/Abstract] OR Non-vitamin K antagonist oral anticoagulants[Title/Abstract] OR NOACs[Title/Abstract] OR direct oral anticoagulants[Title/Abstract] OR DOACs[Title/Abstract] OR novel oral anticoagulants[Title/Abstract] OR new oral anticoagulants[Title/Abstract] OR factor Xa inhibitors[Title/Abstract] OR factor IIa inhibitors[Title/Abstract]  AND TAVI[Title/Abstract] OR TAVR[Title/Abstract] OR transcatheter aortic valve replacement[Title/Abstract] OR transcatheter aortic valve implantation[Title/Abstract] OR aortic valve replacement[Title/Abstract] OR transfemoral aortic valve replacement[Title/Abstract] OR transfemoral valve implantation[Title/Abstract] | 86 |
| EMBASE | 'dabigatran':ti,ab,kw OR 'Pradaxa':ti,ab,kw OR 'rivaroxaban':ti,ab,kw OR 'Xarelto':ti,ab,kw OR 'apixaban':ti,ab,kw OR 'Eliquis':ti,ab,kw OR 'edoxaban':ti,ab,kw OR 'Savaysa':ti,ab,kw OR 'Non-vitamin K antagonist oral anticoagulants':ti,ab,kw OR 'NOACs':ti,ab,kw OR 'direct oral anticoagulants':ti,ab,kw OR 'DOACs':ti,ab,kw OR 'novel oral anticoagulants':ti,ab,kw OR 'new oral anticoagulants':ti,ab,kw OR 'factor Xa inhibitors':ti,ab,kw OR 'factor IIa inhibitors':ti,ab,kw AND 'TAVI':ti,ab,kw OR 'TAVR':ti,ab,kw OR 'transcatheter aortic valve replacement':ti,ab,kw OR 'transcatheter aortic valve implantation':ti,ab,kw OR 'aortic valve replacement':ti,ab,kw OR 'transfemoral aortic valve replacement':ti,ab,kw OR 'transfemoral valve implantation':ti,ab,kw AND [embase]/lim NOT ([embase]/lim AND [medline]/lim) | 97 |
| COCHRANE | (dabigatran):ti,ab,kw OR (Pradaxa):ti,ab,kw OR (rivaroxaban):ti,ab,kw OR (Xarelto):ti,ab,kw OR (apixaban):ti,ab,kw OR (Eliquis):ti,ab,kw OR (edoxaban):ti,ab,kw OR (Savaysa):ti,ab,kw OR (Non-vitamin K antagonist oral anticoagulants):ti,ab,kw OR (NOACs):ti,ab,kw OR (direct oral anticoagulants):ti,ab,kw OR (DOACs):ti,ab,kw OR (novel oral anticoagulants):ti,ab,kw OR (new oral anticoagulants):ti,ab,kw OR (factor Xa inhibitors):ti,ab,kw OR (factor IIa inhibitors):ti,ab,kw AND (TAVI):ti,ab,kw OR (TAVR):ti,ab,kw OR (transcatheter aortic valve replacement):ti,ab,kw OR (transcatheter aortic valve implantation):ti,ab,kw OR (aortic valve replacement):ti,ab,kw OR (transfemoral aortic valve replacement):ti,ab,kw OR (transfemoral valve implantation):ti,ab,kw | 57 |
| Overall |  | 240 |
| Duplication |  | 57 |

Supplementary Table 2. The criteria for outcomes definition.

| **Study (year)** | **Definition of major, life-threatening, or minor bleeding** | **Definition of composite endpoint** |
| --- | --- | --- |
| ENVISAGE-TAVI 2021 | ISTH criteria | Composite of all-cause mortality, myocardial infarction, ischemic stroke, systemic thromboembolic event, valve thrombosis, or major bleeding |
| Didier et al.2021 | NR | NR |
| Kawashima et al.2020 | VARC-II criteria | NR |
| Mannacio et al.2020 | NR | NR |
| Kalogeras et al.2019 | BARC criteria | NR |
| Jochheim et al.2019 | BARC criteria | Composite of all-cause mortality, major or life-threatening bleeding, myocardial infarction, and any clinically relevant cerebrovascular event |
| Kosmidou et al.2019 | NR | NR |
| Butt et al.2019 | NR | NR |
| Mangner et al.2018 | VARC-II criteria | Composite of all-cause mortality, all stroke, life-threatening bleeding, acute kidney injury stage 2 and 3, coronary obstruction requiring intervention, major vascular complication, and valve-related dysfunction requiring repeat procedure |
| Geis et al.2018 | VARC-II criteria | Composite of all-cause mortality, stroke, embolism, severe bleeding |
| Seeger et al. 2017 | BARC criteria | Composite of all-cause mortality, all stroke, life-threatening bleeding, acute kidney injury, coronary obstruction, major vascular complications, and valve dysfunction requiring reintervention |

ENVISAGE-TAVI, Compare the efficacy and safety of edoxaban with vitamin K antagonists in patients with atrial fibrillation as the indication for oral anticoagulation after successful transcatheter aortic valve replacement; ISTH, International Society on Thrombosis and Haemostasis; VARC-II, Valve Academic Research Consortium-2; BARC, Bleeding Academic Research Consortium.

Supplementary Table 3. Demographics and clinical characteristics of the included studies.

| **Characteristics** | **ENVISAGE-TAVI 2021** | **Didier et al. 2021** | **Kawashima et al. 2020** | **Mannacio et al. 2020** | **Kalogeras et al. 2019** | **Jochheim et al. 2019** | **Kosmidou et al. 2019** | **Butt et al. 019** | **Mangner et al. 2018** | **Geis et al. 2018** | **Seeger**  **et al. 2017** |
| --- | --- | --- | --- | --- | --- | --- | --- | --- | --- | --- | --- |
| **Total number (n)** | 1426 | 2471 | 403 | 1032 | 217 | 962 | 933 | 735 | 298 | 326 | 272 |
| **Age (year)** | 82.1 | 83.4 | 84.4 | 68.8 | 82.2 | 81.9 | 82.8 | 82.3 | 80.0 | 83 | 81.3 |
| **Female (%)** | 47.5 | 47.7 | 66.7 | 41.5 | 41.5 | 52.5 | 34.4 | 38.4 | 55.0 | 52.8 | 49.3 |
| **BMI (kg/m^2^)** | 27.7 | 27.0 | 22.2 | 23.2 | 26.6 | 26.5 | 28.4 | NR | 27.7 | 26.8 | 27.3 |
| **Hypertension (%)** | 91.4 | NR | 76.2 | 30.4 | NR | 89.6 | 91.7 | 88.2 | NR | NR | NR |
| **Diabetes mellitus (%)** | 37.0 | 23.1 | 24.3 | 16.4 | 25.5 | 32.3 | 35.3 | 22.3 | 42.3 | 31.9 | 32.4 |
| **Chronic kidney disease (%)** | NR | NR | 75.9 | 11.2 | NR | 47.4 | NR | 11.7 | 31.9 | NR | 46.7 |
| **NYHA class > III (%)** | 45.0 | 68.9 | 56.8 | NR | 81.6 | 78.3 | NR | NR | 72.5 | NR | NR |
| **History of stroke/TIA (%)** | 16.8 | 12.2 | 14.4 | 6.7 | NR | NR | 22 | 18 | 16.1 | NR | NR |
| **History of CAD (%)** | 41.4 | NR | 30 | NR | NR | 47.5 | 20.2 | NR | 42.3 | NR | 62.5 |
| **History of MI (%)** | 13.9 | NR | 6.9 | 12.0 | 12.7 | 14.4 | NR | NR | 9.1 | NR | NR |
| **Prior CABG (%)** | 8.9 | 8.3 | 5.7 | NR | NR | NR | NR | NR | 10.1 | NR | NR |
| **Prior PCI (%)** | 25.8 | 27.2 | NR | NR | 25.4 | NR | NR | NR | 18.8 | NR | NR |
| **CHA_2_DS_2_-VASc *** | 4.5 | NR | 5.1 | NR | NR | NR | 5.6 | 4.9 | 5.0 | 4.7 | 5.0 |
| **HAS-BLED §** | NR | NR | 2.7 | 1.7 | NR | NR | NR | 3.3 | 3.0 | 2.8 | 3.2 |
| **Valve type (%)** | | | | | | | | | | | |
| Balloon-expandable | 47.5 | 67.3 | NR | NR | 25.7 | NR | NR | NR | 35.9 | NR | NR |
| Self-expandable | 45.8 | 31.3 | NR | NR | 65.0 | NR | NR | NR | 64.4 | NR | NR |
| **Antiplatelet treatment (%)** | | | | | | | | | | | |
| Aspirin | NR | 45.6 | NR | NR | 17.3 | NR | NR | NR | NR | NR | NR |
| Clopidogrel | NR | 8.3 | NR | NR | 55.0 | NR | NR | NR | NR | NR | NR |
| DAPT | NR | 9.8 | NR | NR | 26.9 | NR | NR | 16.7 | NR | NR | NR |

*****The CHA2DS2-VASc score was derived from congestive heart failure, hypertension, age ≥ 75 years (2 points), diabetes, history of stroke (2 points), vascular disease, age 65 to 74 years, and female sex. §The HAS-BLED score was calculated by adding 1 point for each of the following parameters: hypertension, abnormal renal/liver dysfunction, history of stroke, history of bleeding or predisposition to bleeding, labile international normalized ratio, age >65 years, and drug consumption of antiplatelet agents, nonsteroidal anti-inflammatory drugs, or alcohol abuse. BMI, Body-mass index; TIA, transient ischemic attack; MI, myocardial infarction; CAD, coronary artery disease; NYHA, New York Heart Association; DAPT, dual antiplatelet therapy; NR, not reported. ENVISAGE-TAVI, compare the efficacy and safety of edoxaban with vitamin K antagonists in patients with atrial fibrillation as the indication for oral anticoagulation after successful transcatheter aortic valve replacement.

Supplementary Table 4. Quality assessment of randomized [controlled](javascript:;) trials.

| **Study** | **Random sequence generation** | **Allocation concealment** | **Blinding of participants and personnel** | **Blinding of outcome assessment** | **Incomplete outcome data** | **Selective reporting** | **Other bias** |
| --- | --- | --- | --- | --- | --- | --- | --- |
| ENVISAGE-TAVI 2021 | Low | Low | High | Low | Low | Low | Low |

Low, low risk; High, high risk.

Supplementary Table5. Quality assessment of real-world studies

| **Study** | **Selection** | **Comparability** | **Outcome or exposure** | **NOS score** |
| --- | --- | --- | --- | --- |
| Didier et al. 2021 | 3 | 2 | 3 | 8 |
| Kawashima et al. 2020 | 3 | 2 | 3 | 8 |
| Mannacio et al. 2020 | 3 | 2 | 3 | 8 |
| Kalogeras et al. 2019 | 3 | 2 | 3 | 8 |
| Jochheim et al. 2019 | 3 | 2 | 3 | 8 |
| Kosmidou et al. 2019 | 3 | 1 | 3 | 7 |
| Butt et al. 2019 | 4 | 2 | 3 | 9 |
| Mangner et al. 2018 | 3 | 1 | 2 | 6 |
| Geis et al. 2018 | 3 | 1 | 2 | 6 |
| Seeger et al. 2017 | 3 | 1 | 3 | 7 |

NOS, NEW-Castle Ottawa scale; The summary risk of bias was determined as low (NOS scores≥7), moderate (4≤NOS scores≤6), and high (NOS score ≤ 3).

Supplementary Table 6. Sensitivity analysis by omitting each study.

| **Study omitted** | **aHR (95%CI)** |
| --- | --- |
| **All-cause mortality** | |
| ENVISAGE-TAVI 2021 | 0.96 (0.63, 1.47) |
| Didier et al. 2021 | 1.00 (0.68, 1.47) |
| Kawashima et al. 2020 | 1.02 (0.69, 1.51) |
| Kalogeras et al. 2019 | 0.94 (0.63, 1.40) |
| Jochheim et al. 2019 | 0.90 (0.59, 1.38) |
| Kosmidou et al. 2019 | 0.96 (0.64, 1.46) |
| Butt et al. 2019 | 0.95 (0.62, 1.45) |
| Mangner et al. 2018 | 1.00 (0.68, 1.46) |
| Geis et al. 2018 | 0.93 (0.62, 1.39) |
| Seeger et al. 2017 | 0.86 (0.71, 1.05) |
| Combined | 0.95 (0.65, 1.39) |
| **Stroke** | |
| ENVISAGE-TAVI 2021 | 0.91 (0.47, 1.76) |
| Mannacio et al. 2020 | 1.03 (0.70, 1.49) |
| Kosmidou et al. 2019 | 0.69 (0.47, 1.01) |
| Mangner et al. 2018 | 0.89 (0.54, 1.48) |
| Geis et al. 2018 | 0.79 (0.52, 1.22) |
| Seeger et al. 2017 | 0.87 (0.53, 1.42) |
| Combined | 0.86 (0.55, 1.34) |
| **Bleeding** | |
| ENVISAGE-TAVI 2021 | 0.76 (0.57, 1.02) |
| Didier et al. 2021 | 0.87 (0.62, 1.24) |
| Kawashima et al. 2020 | 0.86 (0.62, 1.19) |
| Mannacio et al. 2020 | 0.92 (0.70, 1.20) |
| Kalogeras et al. 2019 | 0.81 (0.59, 1.11) |
| Jochheim et al. 2019 | 0.82 (0.57, 1.18) |
| Kosmidou et al. 2019 | 0.81 (0.59, 1.11) |
| Butt et al. 2019 | 0.81 (0.58, 1.12) |
| Mangner et al. 2018 | 0.86 (0.60, 1.23) |
| Geis et al. 2018 | 0.79 (0.58, 1.08) |
| Seeger et al. 2017 | 0.86 (0.63, 1.19) |
| Combined | 0.83 (0.61, 1.13) |
| **Composite endpoint** | |
| ENVISAGE-TAVI 2021 | 1.05 (0.78, 1.42) |
| Jochheim et al. 2019 | 1.04 (0.78, 1.39) |
| Mangner et al. 2018 | 1.09 (0.93, 1.28) |
| Geis et al. 2018 | 1.03 (0.85, 1.25) |
| Seeger et al. 2017 | 1.03 (0.86, 1.22) |
| Combined | 1.05 (0.88, 1.24) |

CI, confidence interval; ENVISAGE-TAVI, compare the efficacy and safety of edoxaban with vitamin K antagonists in patients with atrial fibrillation as the indication for oral anticoagulation after successful transcatheter aortic valve replacement.

Supplementary Table 7. Data of meta-regression of covariates.

| **Variable** | **P value for all-cause mortality** | **P value for stroke** | **P value for bleeding** | **P value for** **composite endpoint** |
| --- | --- | --- | --- | --- |
| Mean age | 0.539 | 0.932 | 0.982 | 0.747 |
| Female | 0.729 | 0.855 | 0.600 | 0.664 |
| BMI | 0.538 | 0.886 | 0.765 | 0.935 |
| Hypertension | 0.656 | 0.618 | 0.638 | 0.753 |
| Diabetes mellitus | 0.633 | 0.648 | 0.357 | 0.972 |
| Chronic kidney disease | 0.601 | 0.997 | 0.391 | 0.770 |
| NYHA > II | 0.787 | 0.725 | 0.655 | 0.840 |
| History of stroke/TIA | 0.859 | 0.544 | 0.687 | 0.742 |
| History of CAD | 0.197 | 0.854 | 0.520 | 0.652 |
| History of MI | 0.444 | 0.623 | 0.472 | 0.755 |
| CHA2DS2-VASc * | 0.931 | 0.829 | 0.642 | 0.489 |
| HAS-BLED § | 0.404 | 0.668 | 0.858 | 0.993 |

*****The CHA2DS2-VASc score was derived from congestive heart failure, hypertension, age ≥ 75 years (2 points), diabetes, history of stroke (2 points), vascular disease, age 65 to 74 years, and female sex. §The HAS-BLED score was calculated by adding 1 point for each of the following parameters: hypertension, abnormal renal/liver dysfunction, history of stroke, history of bleeding or predisposition to bleeding, labile international normalized ratio, age >65 years, and drug consumption of antiplatelet agents, nonsteroidal anti-inflammatory drugs, or alcohol abuse. BMI, Body-mass index; NYHA, New York Heart Association; TIA, transient ischemic attack; CAD, coronary artery disease; MI, myocardial infarction.

**
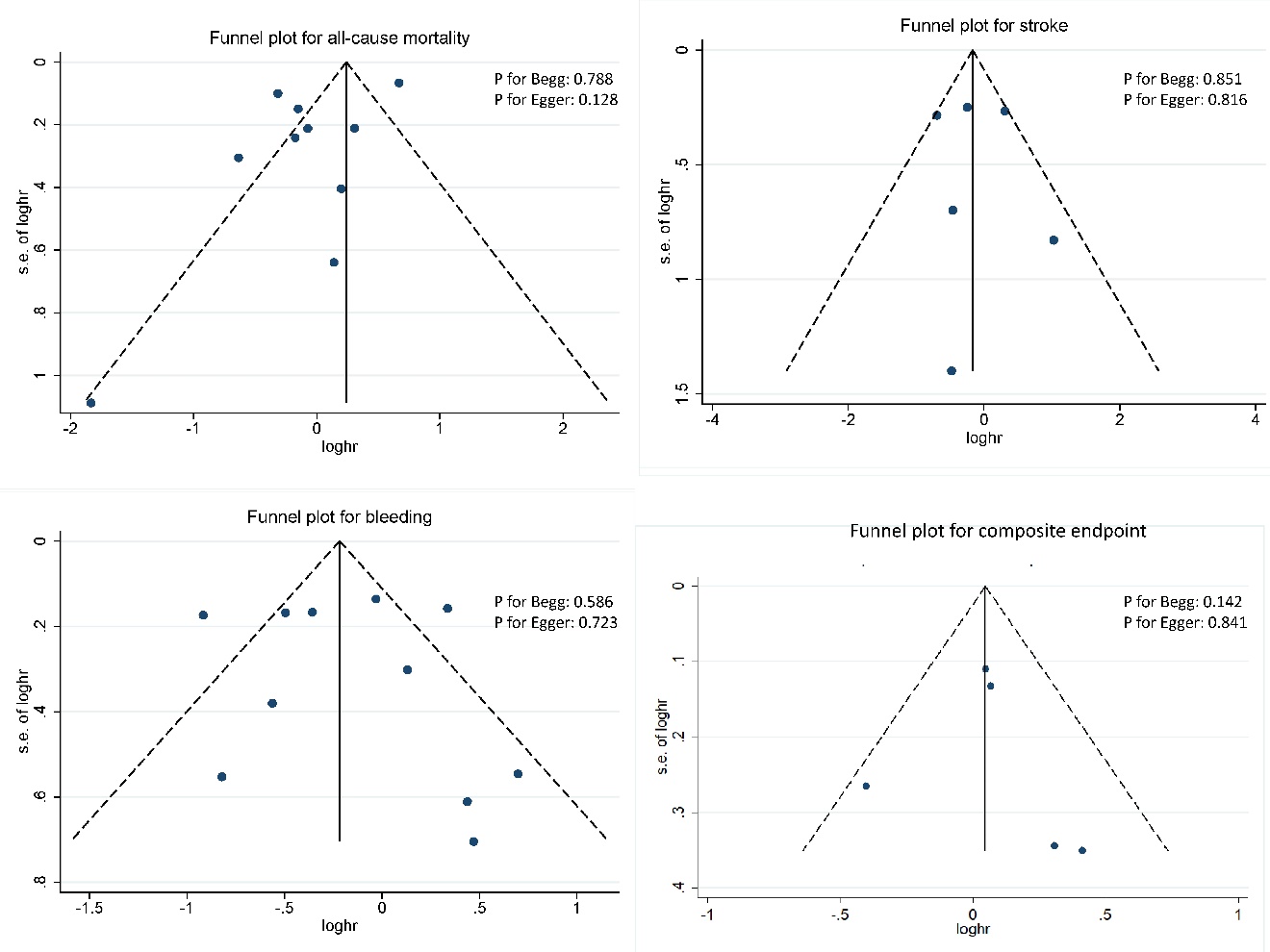
**

Supplementary Fig. 1. Funnel-plot and test for publication bias.
